# Fibrous Microplastics Release from Textile Production Phases: A Brief Review of Current Challenges and Applied Research Directions

**DOI:** 10.3390/ma18112513

**Published:** 2025-05-27

**Authors:** Md Imran Hossain, Yi Zhang, Abu Naser Md Ahsanul Haque, Maryam Naebe

**Affiliations:** Institute for Frontier Materials, Deakin University, 75 Pigdons Road, Geelong, VIC 3216, Australia; md.i.hossain@deakin.edu.au (M.I.H.); yi.zhang@deakin.edu.au (Y.Z.); a.haque@deakin.edu.au (A.N.M.A.H.)

**Keywords:** microplastic pollution, textile production, recycle and upcycle, dry and wet processes, sustainability

## Abstract

Microplastics (MPs), particularly fibrous MPs, have emerged as a significant environmental concern due to their pervasive presence in aquatic and terrestrial ecosystems. The textile industry is a significant contributor to MP pollution, particularly through the production of synthetic fibers and natural/synthetic blends, which release substantial amounts of fibrous MPs. Among the various types of MPs, fibrous MPs account for approximately 49–70% of the total MP load found in wastewater globally, primarily originating from textile manufacturing processes and the domestic laundering of synthetic fabrics. MP shedding poses a significant challenge for environmental management, requiring a comprehensive examination of the mechanisms and strategies for the mitigation involved. To address the existing knowledge gaps regarding MP shedding during the textile production processes, this brief review examines the current state of MP shedding during textile production, covering both dry and wet processes, and identifies the sources and pathways of MPs from industrial wastewater treatment plants to the environment. It further provides a critical evaluation of the existing recycling and upcycling technologies applicable to MPs, highlighting their current limitations and exploring their potential for future applications. Additionally, it explores the potential for integrating sustainable practices and developing regulatory frameworks to facilitate the transition towards a circular economy within the textile industry. Given the expanding application of textiles across various sectors, including medical, agricultural, and environmental fields, the scope of microplastic pollution extends beyond conventional uses, necessitating urgent attention to the impact of fibrous MP release from both synthetic and bio-based textiles. This brief review consolidates the current knowledge and outlines the critical research gaps to support stakeholders, policymakers, and researchers in formulating effective, science-based strategies for reducing textile-derived microplastic pollution and advancing environmental sustainability.

## 1. Introduction

Microplastics (MPs), originating from the degradation of larger plastic materials, are prevalent pollutants across various environments, including oceans [1,2], rivers and lakes [3], Arctic Sea ice [4], marine [5], and freshwater sediments [6], sewage sludge, and agricultural soils [7]. One of the major components of MPs is the synthetic microfibers or fiber-shaped microplastics released from textiles [8]. They are released throughout the textile lifecycle, including the production, usage, maintenance, and disposal phases [9,10]. The accumulation of fiber-shaped MPs in the environment [2] is expected to worsen in the upcoming years due to the growing fast-fashion trend [11], favored by the slow degradation rate of the released MPs and the absence of any recycling methods. Additionally, the degradation capability of natural fibers (such as cotton and wool) can also be delayed by their strong attachment to synthetic dyeing and finishing agents, further contributing to MP accumulation [12]. Moreover, the wide use of natural/synthetic blends in diverse fields accelerates synthetic fiber release, while emerging bio-based synthetic polymers in textiles, such as polylactic acid (PLA), often need industrial composting to degrade. Therefore, the release of such bio-derived and naturally non-degradable fibers poses similar hazards to the environment.

**Figure 1:**
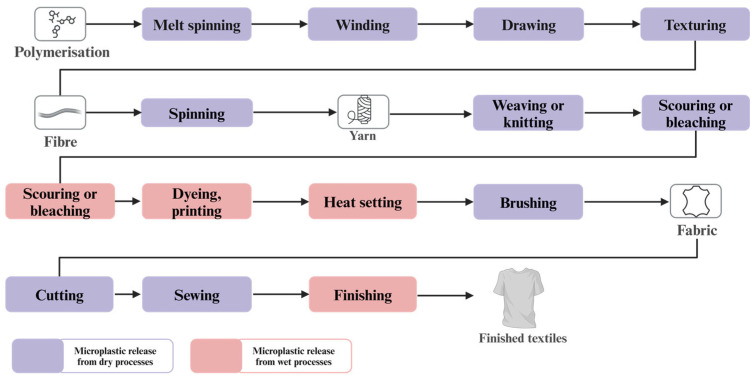
Key methods in synthetic textile production reveal distinct stages within both dry and wet processing, where microplastic shedding is most prevalent during manufacturing.

Synthetic textile manufacturing or textiles from bio-derived polymers typically start with polymer chips, which are melted and extruded into continuous filaments. Filaments are initially chopped into shorter staple fibers before undergoing carding, a mechanical process that separates, cleans, and blends them into a uniform sliver. This sliver, a loosely organized strand of fibers, acts as a crucial precursor in yarn production, preparing the material for spinning or weaving. Before spinning, filaments may undergo a texturing process to impart desirable properties such as increased bulk, elasticity, and a more natural feel, such as introducing loops, crimps, or coils into the filaments, enhancing their texture and appearance. Next, these slivers undergo various yarn-spinning techniques to produce yarn. Yarns made from short staple fibers are known as “spun yarns”, whereas those made from continuous filaments are called “filament yarns”. These yarns are subsequently woven or knitted into textiles with a few additional steps, such as pre-treatment, dyeing, printing, finishing, and rinsing [13]. Woven textiles are formed by the perpendicular interlacing of two threads, while knitted textiles involve a single thread looping symmetrically along a course [14]. The manufacturing process of synthetic fabrics can be broadly categorized into two main stages: dry processing and wet processing, as illustrated in Figure 1. However, it is important to note that some variations and alternatives are also employed. Among the various stages of synthetic fiber processing for textile manufacturing, there is a notable risk of releasing MPs into the environment. This risk primarily arises during processes such as cutting synthetic filaments into shorter staple fibers and subsequent dry and wet textile manufacturing processes. These processes result in the shedding of MPs in significant quantities.

MPs from textiles are more likely to be in a fibrous shape, which is known to be more harmful than other common MP shapes, such as spherical or fragmented, due to the way that they pass through the gut [15]. The term “microplastic” does not necessarily differentiate MPs from textiles or other sources, though they require distinct attention. Our group has earlier provided standard terminology to be used for the MPs released from textiles identifiable by a length-to-diameter ratio of at least 3:1 and categorized by length, i.e., fibrous nanoplastic (3–3000 nm), fibrous microplastic (3–3000 µm), and fibrous mesoplastic (3–15 mm) [15]. This is supported by numerous early reports that confirmed the unique shape of textile MPs that are different from all other kinds.

It is challenging to determine the exact number of non-degradable textiles consumed every year, although their environmental impact is plausible from the high production rate of textiles and consumer demand. Classifying the percentage contribution of textile-originated MPs within the overall presence of MPs in the environment poses significant challenges due to ongoing controversies. A recent study indicates that a substantial number of fibrous MPs are shed from synthetic textiles released from the production phases of textiles [16]. Some of the fibers from the production processes are captured in wastewater treatment plants (WWTPs), and the retained semi-solid sludge goes directly to landfills or agricultural fields.

Textile fibers undergo extensive processing involving heavy machinery and intense thermochemical treatments during the production phases, which are likely to result in a significant release of fibrous MPs. Identifying the sources of MP shedding during the production stages of the textile industry has proven to be a major challenge. Most existing reviews on textile MPs, however, primarily concentrate on the release of fibrous MPs during post-production stages [17] and through consumer use [18], such as during laundering [19]. Therefore, it is essential to conduct a systematic review and evaluate the recent findings to identify the sources of MP shedding in textile bulk production. Parallelly, it is also crucial to pinpoint the available recycling and upcycling technologies for MPs and their limitations to reduce their environmental impact and spur innovation and economic opportunities.

To conduct this brief review, the Web of Science database was searched for peer-reviewed literature on textile microplastics in May 2025. The search terms Topic = (“microplastics”) AND (“textiles”) were applied, with the document type limited to “Article” and “Review”. This search yielded 747 articles, of which approximately 500 were published since 2021—highlighting the growing attention to the environmental hazards posed by textile-derived MPs. To ensure comprehensive coverage and validate our findings, we also conducted a comparable search using other well-regarded academic databases, such as Scopus. The results obtained from these platforms consistently reflected similar trends in both publication volume and thematic focus. However, as noted above, the majority of these studies focused on the post-production phases, while MP release during the bulk production processes (involving heavy thermo-mechanical treatments) remains significantly underexplored. Further filtering based on three specific criteria, namely (1) research addressing MP release during textile manufacturing processes, (2) studies involving wastewater treatment in textile industries, and (3) investigations into recycling or upcycling of textile-derived MPs, reduced the pool to only around 20 relevant publications.

Given this substantial knowledge gap, a brief review was undertaken to consolidate the limited but emerging research on MP shedding during the textile production stages. These upstream sources are critical yet often overlooked contributors to microfiber pollution. By highlighting this underrepresented area, the brief review aims to raise awareness, identify the key research gaps, and support the development of informed mitigation strategies. To the best of our knowledge, this is the first review that explores the sources of MP shedding during textile bulk production, covering both dry and wet processes, and highlights the potential for recycling and upcycling. In the present brief review, and when reporting data, the term “MPs” is mainly defined to refer to synthetic fibrous plastics with a length from nanoplastics (3–3000 nm) to microplastics (3–3000 µm) released from textiles. The mesoplastics (3–15 mm) were excluded from our scope, as they are typically larger, more visible, and can be easily captured through conventional filtration methods. The topics covered in this brief review provide a foundational framework for research on mitigating MP shedding during textile production. Additionally, the brief review seeks to provide insights that can guide sustainable manufacturing practices, regulatory frameworks, and innovation in textile recycling technologies.

## 2. Microplastic Shedding from the Textile Production Process

Textile industries significantly contribute to MP pollution by shedding fibers across various stages of production, including dry processes such as melt spinning, winding, drawing, texturing, spinning, weaving or knitting, heat setting, brushing, cutting, sewing, and wet processes such as scouring, bleaching, dyeing, printing, and finishing.

**Figure 2:**
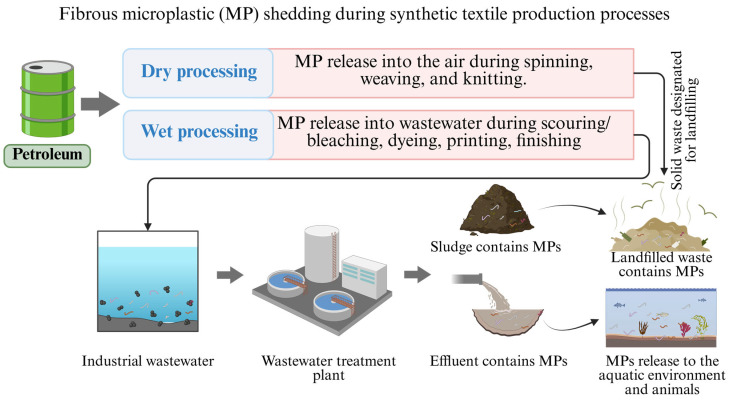
Schematic illustration of fibrous MP release pathways in textile production. The diagram shows details of shedding from dry and wet processing, subsequent pathways through WWTPs, sludge, and eventual dissemination into the environment.

Figure 2 illustrates the relationship between the stages of the textile production process and MP shedding. It highlights the points at which MPs are released during both dry and wet processing and their primary pathways into the environment through air, sludge, and wastewater. The following subsections further detail these pathways.

### 2.1. Factors Influencing Microplastic Shedding During Textile Dry Processes

Throughout the textile dry processes [20,21], including spinning [22], weaving, and knitting [23], shedding of MPs can occur [24]. The initial stage of synthetic fiber production (such as melt spinning) can release MP particles or fibers [25], particularly if the machinery used is not well maintained or if there is friction between the filaments and equipment. After the filaments are drawn, they may undergo texturing, a process that introduces a crimp or curl to the fibers to improve their elasticity and bulk. Texturing involves heat and mechanical action, both of which can contribute to the shedding of MPs, especially when fibers are stretched and manipulated. The process varies for acrylic fibers as they are produced through wet-spinning processes [26], though the method of fiber processing, including stretching and manipulating for texturizing or introducing a crimp or curl, is similar.

The texturized filaments are often cut into shorter staple fibers to blend with natural fibers. Spinning, particularly when short fibers are involved, is a major source of MP shedding. As fibers are twisted and handled, loose microfibers are generated and become airborne, settling in the surrounding environment. Studies indicate that the mechanical stress involved in dry processes leads to fiber breakage and detachment [14]. Spinning and various processes of spinning methods, such as ring, compact, and air-jet, significantly contribute to the shedding of MPs [27]. Table 1 shows recent studies indicating that the air-jet spinning process results in relatively low MP shedding during washing, with reported values as low as 16 ± 2 mg/kg, compared to significantly higher values such as 1709 ± 28 mg/kg observed for rotor spinning. This trend is consistently observed throughout most studies, although some exceptions have been reported. Consequently, a substantial portion of MP pollution originates from shedding during the mechanical treatment of fibers and fabrics.

**Figure 3:**
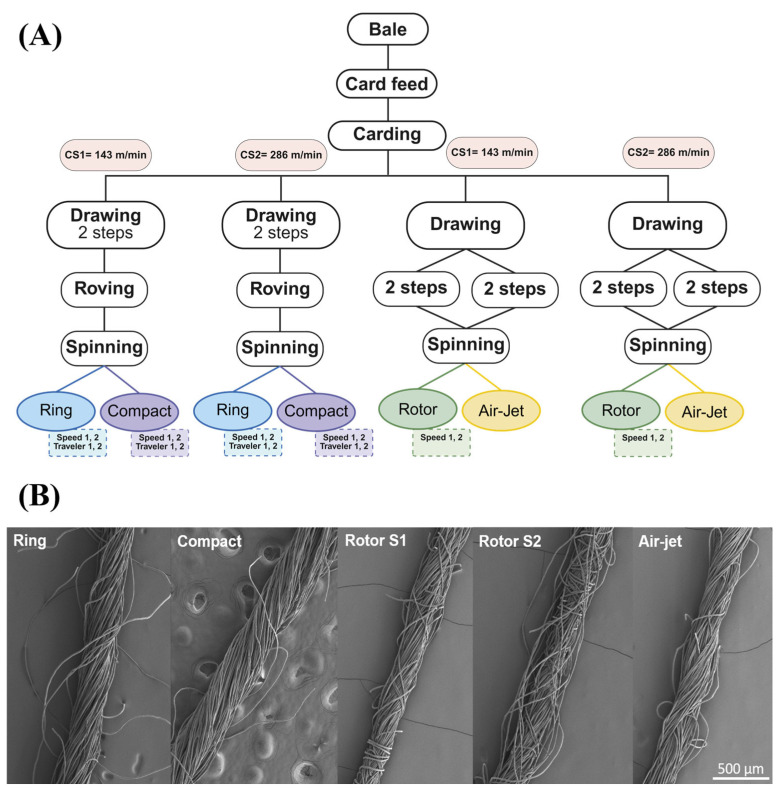
(**A**) Schematic diagram of yarn production at different speeds with different colors for different mechanisms. (**B**) Scanning electron microscope (SEM) images of yarns produced using different spinning methods. Adapted from [27] with permission from Elsevier.

Further, yarn hairiness and lint generation differ based on the yarn type and the knitting process that they are designed for. For instance, the production of fibrous MPs is greater in ring-spun yarns than in compact and rotor-spun yarns. A recent study detected MPs in all samples, with concentrations ranging from 44 to 8057 MPs/g. Rotor-spun yarns exhibited a high content of MPs, ranging from 2000 to 8000 MPs/g as a result of the wash. In contrast, other samples, including yarns produced by alternative spinning methods, showed fiber counts in the range 10–1000 MPs/g [27] (Figure 3).

Factors such as yarn type, pre-treatment, and fiber characteristics influence fiber shedding variability. Understanding and mitigating these sources of MP release is essential for reducing the environmental impact and enhancing the sustainability of textile production in the dry processes.

Once the yarn is produced, it is used in either weaving or knitting to form textiles. The processes apply mechanical forces to the yarns, which dislodge fibers, especially when staple fibers are used. In the dry-finishing phase of textile production, various mechanical treatments, such as brushing and sanding, are applied to the fabric to provide specific textures, such as fleece. These surface treatments involve vigorous mechanical action, which generates large amounts of loose fibers. For example, fleece textiles are known to shed MPs at a high rate during production and consumer washing [31]. The mechanical forces applied during these finishing processes are key contributors to MP shedding, as fibers are dislodged from the textile surface. After finishing, the cutting and sewing stages also contribute to MP shedding, particularly when synthetic fabrics are used [20].

Another critical stage in synthetic textile production is heat setting, where fabrics are exposed to elevated temperatures to stabilize their shape and dimensions. While heat setting enhances the performance of synthetic fabrics, it also contributes to fiber degradation, particularly if the fibers become weak during the earlier stages of the production process.

The presence of microfibers in the air and atmospheric transport has garnered growing attention [9,32]. For example, a study in Hamburg, Germany, reported a significant abundance of microplastics in the atmospheric fallout within the metropolitan area [33]. The inhalation of airborne MPs poses a greater risk for human exposure compared to other pathways. These MPs can be directly inhaled, while settled particles may be ingested through hand-to-mouth activities, particularly by children [34]. MPs have been identified in human stools, serving as direct evidence of widespread exposure. Inhalation of airborne MPs has been linked to negative health outcomes [35], including chronic obstructive pulmonary disease [36]. Recent studies on indoor and outdoor MP pollution have revealed alarming data regarding the concentration of MPs in the air (Table 2). The concentration of MPs in outdoor environments can be as high as 365 ± 69 MPs/day/m^2^, with a substantial proportion originating from textile sources [37]. Synthetic textiles in closed conditions can release up to 403 ± 65 MPs/g of fabric [38].

Airborne MPs released during textile dry processes present significant health risks to textile workers, particularly those who handle synthetic fibers such as polyester, nylon, and acrylic. The inhalation of MPs, which are released during different production processes including spinning, weaving, dyeing, and finishing, can lead to respiratory problems such as coughing, breathlessness, and diminished lung function. These conditions may escalate to chronic inflammation and severe diseases like asthma [39] and lung cancer [40]. Research has shown that MP concentrations are markedly higher in indoor environments of textile factories compared to outdoor settings, thereby increasing the exposure risk for workers [41]. Furthermore, inhaled MPs can migrate from the lungs to other organs, potentially causing cardiovascular, renal, and neurological disorders, and can also act as vectors for toxic substances [42]. Long-term exposure effects have also been documented, including impaired lung development and potential risks of fatal development in pregnant workers [43]. To mitigate these health risks, it is crucial to enforce stricter air quality regulations, enhance factory ventilation systems, and provide more effective protective equipment to ensure the safety of workers in the textile industry.

**Table 2:** Concentration, distribution, and type of MPs in indoor and outdoor atmospheric environments.

Conditions	Composition/Type of Fabric	Size and/or Amount	Reference
Outdoor—environment	-	2–100 µm; ~1 MPs/m^3^	[44]
Outdoor—environment	PET, PS, PE, PVC, PET	25–2600 µm; 365 ± 69 MPs/day/m^2^	[37]
Indoor—closed condition	PET-woven-filament	1 ± 1 MPs/g of fabric	[38]
	PET-knit-filament	108 ± 44 MPs/g of fabric	
	PET-Knit-staple	347 ±102 MPs/g of fabric	
	PET/COT-knit staple	403 ± 65 MPs/g of fabric	
Outdoor—dryer exhaust	Polyester	93 ± 17 MPs/g of fabric; 15 min of drying	[45]
	Cotton	72 ± 11 MPs/g of fabric; 15 min of drying	
Indoor—closed condition	Polyester	1.6 ± 1.8 MPs/m^3^	[46]

COT: cotton, PS: polystyrene PE: polyethylene, PVC: polyvinyl chloride.

### 2.2. Factors Influencing Microplastic Shedding During Textile Wet Processes

Apart from the dry processing discussed earlier, wet production processes, such as scouring, bleaching, dyeing, finishing, and washing, significantly contribute to MP shedding. Scouring is aimed at removing natural impurities, such as waxes or oils, from fibers, and, in the case of synthetic textiles, it is for eliminating lubricants or antistatic agents used during spinning [21]. Fabrics are treated with alkaline solutions, such as sodium hydroxide, which weakens the fibers, contributing to MP shedding. The chemicals used during this process can create microfractures in the fibers, leading to fiber disintegration and the release of MPs into the wastewater. Studies indicate that scouring, using neutral soap at 40 °C for polyester, does not significantly affect microfiber shedding across consecutive wash cycles [47]. However, other research shows a gradual decrease in shedding after initial washes due to the release of loose, embedded fibers. This suggests that scouring can reduce future shedding but raises concerns about a high microfiber content in scouring effluent [48]. Following scouring, textiles undergo bleaching, where strong oxidative agents like hydrogen peroxide are applied to whiten the fabric. This step is critical for brightening synthetic fabrics but can further weaken the structure of the fibers. Bleaching not only contributes to direct MP shedding but also weakens the fibers for the subsequent processing stages. Studies show that bleaching weakens synthetic fibers, increasing their fragmentation during washing. De Falco and co-workers found that bleaching synthetic fabrics leads to a higher MP release than unbleached fabrics due to polymer degradation [44]. Rathinamoorthy et al. recommended that further research is needed on bleaches, particularly in conjunction with the effects of fabric softeners [24].

During dyeing, synthetic fabrics are immersed in dye baths containing various chemical solutions that bond to the fibers to achieve the desired color. For materials like polyester and nylon, high temperatures and mechanical agitation are typically used, which exacerbates the fiber release. Dyeing of the fibers involves various synthetic dyes, including reactive, acid, metal complex, and direct dyes [49]. Most textile dyes are intentionally designed to resist mild oxidation and reduction processes to withstand washing cycles and sunlight exposure [50]. These dyes thus remain on the fiber fragments and are released into the environment through hydrolysis and degradation. Consequently, the dye molecules chemically bonded to the fiber surfaces slow down the biodegradation of the fibers. They are also difficult to filter out during wastewater treatment [51]. This is particularly concerning, as untreated dye-laden MPs can persist in aquatic environments, posing risks to both ecosystems and human health.

Printing, a process used to apply patterns or designs to fabric, also contributes to MP shedding. The mechanical action involved in applying dyes or pigments onto fabric surfaces can generate fiber loss, especially when synthetic materials are involved. Both dyeing and printing processes result in significant MP emissions, which are frequently detected in textile industry wastewater [52]. Once the textiles are dyed or printed, they undergo rinsing (or washing), where excess dyes, chemicals, and impurities are washed out from the fabric. While this stage is necessary to achieve color fastness, it also releases MPs into wastewater. The mechanical forces of water jets combined with the chemical residues from earlier treatments contribute to the dislodgement of loose fibers.

**Figure 4:**
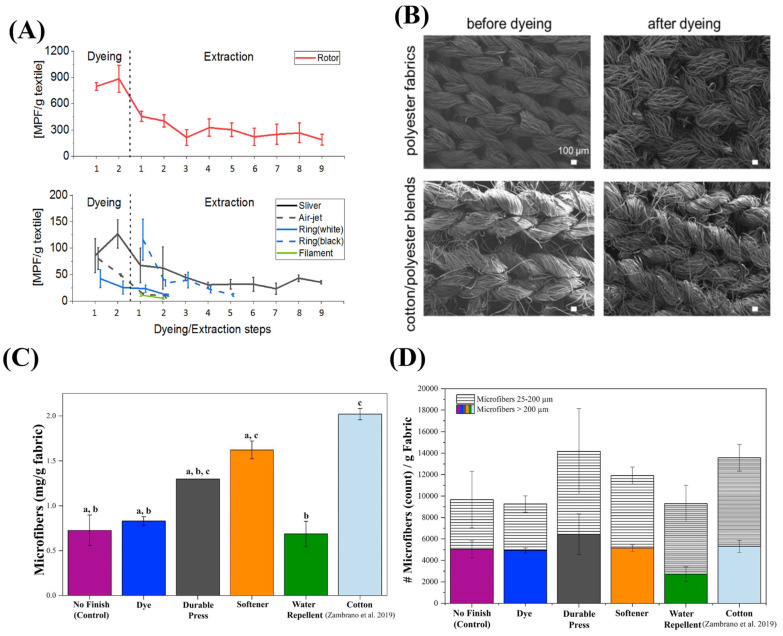
(**A**) Number of MPs per gram of the textile from slivers and yarns, (**B**) SEM images of polyester and cotton/polyester blend fabrics before and after dyeing, (**C**) different fabric finishes impact the number of MPs released during washing; samples sharing the same letter (e.g., a, b; a, b, c; a, c) are not significantly different at a significance level of 0.05, according to the Tukey grouping method for Least Square Means. Samples with different letters (e.g., b; c) are significantly different. (**D**) number of MPs released from fabrics treated with various finishes including no finish (magenta), dye (blue), durable press (dark gray), softener (orange), water repellent (green), and cotton (light blue). Each bar is divided by the length of MPs: the horizontally hatched area represents MPs length range from 25–200 µm, while the solid-colored portion represents the ranges larger than 200 µm. Adapted from [8,13,14] with permission from Elsevier.

During dyeing and printing, all dyed products (sliver, rotor, air-jet, and white-colored ring yarn) exhibit a significant initial increase in MP release, followed by a decrease in subsequent extractions. Despite multiple extractions, the sliver and rotor yarns showed persistently high MP levels, approximately 50 and 220 MPs/g, respectively. In contrast, the air-jet yarn and the white and black ring yarns maintained lower MP levels, less than 20 MPs/g. The filament yarn consistently showed low MP levels (less than 20 MPs/g) throughout the extraction steps. These findings indicate that the dyeing process significantly influences the initial MP release, with variations observed across different yarn types [14] (Figure 4A). Additionally, there is a significant difference in the SEM images for polyester and cotton/polyester blend fabrics when comparing the samples before and after the dyeing process [13]. For polyester fabrics, the SEM images show a relatively smooth surface before dyeing. However, post-dyeing, the surface exhibits an increased roughness and the presence of microcracks, indicating fiber degradation due to the dyeing, using chemicals, and the thermal stress applied during the process. In contrast, the cotton/polyester blend fabrics display a more complex morphology. Before dyeing, the blend shows a combination of smooth polyester fibers and the characteristic rough texture of cotton fibers. After dyeing, the SEM images highlight significant changes, particularly in the cotton fibers, which appear more swollen and roughened. This is likely due to the absorption of dye and water, which causes the cotton fibers to expand and become more susceptible to mechanical damage (Figure 4B).

The final step in the wet production process is finishing, where additional treatments are applied to improve the fabric’s functionality. Different polymeric finishes are commonly used in textile industries to enhance the fabric properties, such as water-repellent [53], flame retardants [54], antimicrobial [55], abrasion-resistant [56], pilling-resistant [57], wrinkle-resistant [58], and UV-protective [59]. The effects of these polymeric finishes and their relation to MP shedding are still unclear. Further, the mechanical action of applying these coatings, combined with the weakening effects of earlier chemical treatments, leads to significant MP shedding. In addition, non-fibrous MP materials like acrylic copolymer and polyester have been found in wash effluents, likely introduced during chemical finishing processes. Ekaterina and her co-workers identified various plastic polymers such as acrylic copolymer, polystyrene, aminoplast resin, and vinyl acetate copolymers in the wash effluent of samples [60]. Many other particles found in effluent water are thought to be introduced during chemical finishing processes. It is suggested that these particles might originate from non-reported chemicals or materials used in textile manufacturing [60]. Some of these coating compounds themselves degrade over time from textiles, contributing to further MP release when the textiles are used or washed by consumers. Table 3 represents the impact of finishing and the dyeing process on MP shedding as reported in the literature. The dyeing process of polyester yarn can result in the release of up to 884 ± 154 MPs per gram of yarn. Additionally, the widely used wrinkle-resistant finishing treatment, known as durable press, can release up to 14,200 microfibers per gram of cotton fabric during washing. In general, textile wet processes significantly impact MP shedding. Along with sensible process control, effective wastewater treatment is crucial to control this impact and prevent the release of MPs into the environment.

## 3. Microplastics in the Industrial Wastewater Treatment Plant

Textile wet processes, particularly dyeing, printing, and finishing segments, are notorious for their significant environmental impact due to their highly polluting nature. These processes not only require substantial water usage but also result in large volumes of wastewater. The detailed chemical composition containing dyes in textile wastewater and their constituent chemicals and environmental impacts [61] is crucial. Additionally, research indicates that textile production can generate up to 200 L of wastewater per kilogram of fabric [62]. The manufacturing steps, including scouring, bleaching, dyeing, and printing, consume most of the water, with usage depending on the machinery, fabrics, and types of dyes involved. Additionally, a considerable amount of dyes and auxiliaries used in these processes are discharged into wastewater, contributing to the environmental burden. Despite the extensive volume of publications related to MPs in literature, most of these studies primarily focus on quantifying the amount of contaminated water released by textile materials and their relationship to the total concentration of MPs in municipal WWTPs [63]. However, industrial WWTPs have received less attention even though many industries have limited or less efficient facilities to remove MPs from the effluent [64,65]. A recent review by Miino and colleagues reported that approximately 49–70% of detected microplastics (MPs) are in fibrous form in wastewater around the world, suggesting that a significant proportion may originate from textile industry processes or the domestic laundering of synthetic fabrics [66]. In many instances, the WWTPs associated with the textile industry are not sufficiently equipped to capture microplastics effectively. Though contrasting results are also reported, i.e., a reduction of 99% in MPs from textile wastewater [67], studies have identified a significantly higher concentration of MPs in the sediments and surface water surrounding industrial areas, particularly those associated with textile production. In a study in China, it was reported that on average, the surface water contained 6.8 MPs/L, while the sediment samples ranged from 16.7 to 1323.3 MPs/kg of dry weight. These concentrations were notably twice as high as those in a reference area used for comparison. Among the MP types generated from all other sources, fibrous MPs were the predominant type, constituting 95% of the surface water samples and 79% of the sediment samples [68]. The analysis revealed that polyester and rayon fibers were the most commonly found types, highlighting the textile industry’s substantial role in contributing to MP pollution. The study also emphasized the critical need for further research into textile effluent and WWTPs in the textile industries [68].

Most studies on MPs and microfiber shedding in the textile industry focus on municipal WWTPs. In 2018, Xu and colleagues were pioneers in documenting the presence of MPs in the effluents of industrial WWTPs associated with textile manufacturing. Their research centered on a WWTP that can treat 30,000 tons of wastewater daily, serving various printing and dyeing facilities within a Chinese textile industry park [69]. Later, a comprehensive review in 2019 mentioned that the average MP abundance ranged between 7.7 and 9.48 MPs/L, with a retention efficiency of 89.17–97.15% in a WWTP. The average length of MPs identified was between 100 and 500 μm [70]. Wang et al. and Zhou et al. investigated MPs in industrial WWTPs linked to the textile industry in China. Both studies reported high removal efficiencies, ranging from 92.8 to 97.4%, yet significant amounts of MPs still reached the aquatic environment. Wang’s study found that MPs in treated effluent were between 8 and 23 MPs/L, while Zhou noted microfiber levels as high as 537.5 MPs/L in WWTPs receiving influents from 130 textile mills, comparable to nearby surface water contamination [71,72]. In a 2021 study, it was reported that the wastewater released from a textile facility producing polyester fabric contained approximately 361 MPs/L [73]. Most of these polyester fibers had an average length of less than 500 µm, and it showed that the capturing of MPs can be effectively reduced by the removal of suspended solids in the liquid [73].

**Figure 5:**
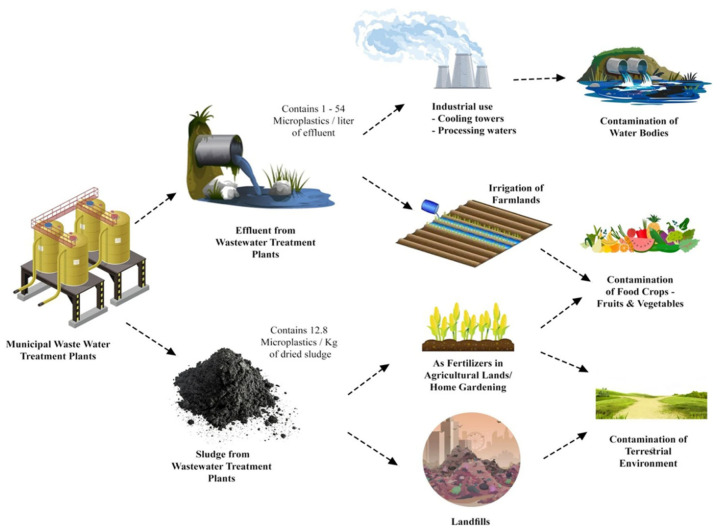
Overall pathway of MP accumulation in the environment from municipal and industrial WWTPs. Adapted from [63] with permission from Springer Nature.

The substantial environmental impact of the textile industry, particularly through wastewater discharge processes, is well documented. These activities significantly contribute to MP pollution. The current facilities and processes often fail to adequately filter out MPs, leading to their continued presence in treated effluents [23]. This shortfall highlights the need for improved filtration technologies and process optimizations to mitigate MP pollution from textile industry discharges. Figure 5 presents a comprehensive overview of the pathways through which fibrous MP pollution enters the environment.

## 4. Recycling, Upcycling Technologies, and Future Directions

Enhancing the recycling and upcycling efficiency of textile fibers can substantially decrease the textile industry’s and its consumers’ waste [74,75]. This improvement minimizes the environmental impact and contributes to a more sustainable production and consumption cycle within the textile sector. However, recent estimates suggest that 75% of the materials used during clothing production and after use are either landfilled or incinerated. Only about 23% of discarded clothing is collected for recycling, with a mere 1% of these recovered fibers being utilized to manufacture new garments, indicating a significant gap in closing the loop through fiber-to-fiber recycling [76,77]. Recent studies have explored several advances in recycling and upcycling technologies for fiber sheds from the textile industry. However, significant limitations remain in effectively recycling and upcycling these fibers, both from industrial processes and consumer use. In open-loop recycling, textile waste is transformed into various new products or applications, while in closed-loop recycling, the waste is processed to produce the same type of product as the original. The diverse types and colors of fibers in most textile waste lead to the production of shoddy fibers, which are considered unattractive and unsuitable for spinning into high-quality textile yarn, thus limiting the effectiveness of closed-loop recycling. In contrast, a few studies have reported the potential for producing high-value products from end-of-life waste textile fabric materials, such as synthetic fibers [78], composites [79], microcrystalline cellulose [80,81], regenerated fibers [82,83], and acoustic insulation [84].

The recycling and upcycling of shed MPs from the textile industry present significant challenges due to the complex nature of these materials and the limitations of current technologies. MPs are tiny fibers, often less than 5 mm in size, contributing to the growing problem of MP pollution in the environment. Addressing this issue requires a comprehensive understanding of the limitations and potential solutions for the recycling and upcycling of these materials. The primary challenges include the mixed material composition and contamination of microfibers, technological constraints, economic viability, and environmental impact. Current recycling technologies are not well suited to handle the small size and mixed composition of MPs, and the costs associated with collecting, cleaning, and processing these fibers can be prohibitively high [9]. Additionally, the recycling process itself can generate secondary waste streams that need to be managed [85]. The upcycling of microfibers presents notable challenges, including maintaining the quality and functionality of the upcycled products, integrating innovative design strategies, gaining market acceptance, and navigating regulatory and standardization hurdles. Addressing these challenges requires advancements in recycling technology, economic incentives, regulatory backing, increased collaboration, and educating consumers [80]. Overcoming these obstacles can lead to a more sustainable and circular approach to managing MP waste. This will help mitigate the textile industry’s environmental footprint and safeguard aquatic ecosystems. Global fabric production has steadily increased over the years, and it is projected to continue this upward trend. The demand for clothing is heavily influenced by fashion trends and styles that frequently shift across different regions and cultures [86]. The extent to which MPs generated during textile production contribute to global MP pollution remains inadequately quantified. Further research is required to accurately determine the proportion of MPs originating from textile manufacturing and their impact on global MP emissions.

## 5. Conclusions

This brief review evaluated the shedding of MPs from textile industries during the production phases. We have reviewed how various processes within textile production, specifically the dry and wet processes, contribute to MP shedding and their impact on industrial WWTPs. We highlighted the processes, such as spinning, weaving, and knitting in the dry phase and bleaching, scouring, dyeing, printing, and finishing in the wet phase, that are most influential in MP shedding. While significant progress has been made in understanding these processes, many mechanisms behind MP shedding remain unexplored, leading to unexpected results. For example, it was initially assumed that a substantial portion of the shed materials from the synthetic textiles consisted of nanoplastics [87]; however, subsequent findings revealed that most were oligomers [88]. Policy gaps exist regarding reducing MP shedding during textile production, underscoring the need for comprehensive strategies as the textile industry is a major source of MP pollution. The continued increase in MP shedding from textiles seems inevitable unless there is a significant advancement in the current technologies. To address this issue, it is critical to examine the several factors within textile manufacturing that contribute to MP shedding. By adopting recycling and upcycling methods for the captured fibers, we can move toward a more sustainable solution. The gaps and limitations in handling MP waste, recycling, and upcycling technologies have been identified for future applications. Exploring the origins of MP shedding in the textile industry and understanding how different manufacturing processes contribute to MP shedding will be key to developing effective strategies to reduce the overall MP pollution. Developing and refining technologies aimed at minimizing MP shedding during textile production presents a promising approach to tackling this pressing environmental issue, ultimately fostering the growth of a more sustainable world.

## Figures and Tables

**Table 1:** Influence of different spinning methods for shedding MPs during washing.

Type of Fiber	Spinning Method	Size/Weight of the Fiber Washed	Washing Parameters [Volume; Duration; Temp.; Cycles]	Detergent	Weight of MPs [mg/kg] or Percentile Released	Average Length of MPs [µm]	References
PET staple fiber	Ring	1.5 ± 0.1 g (1 ktex = 1 g/m, yarn)	150 mL; 45 min; 40 °C; 3 cycles	LAS; 50 mL	66 ± 21	528 ± 66	[27]
	Compact				73 ± 19	575 ± 115	
	Rotor				1709 ± 28	216 ± 1	
	Air-jet				16 ± 2	328 ± 39	
PET staple fiber	Ring	200 × 340 mm^2^; folded fabric	350 mL; 45 min; 40 °C; 5 cycles	AATCC (reference detergent)	~940	~520	[28]
	Rotor				~630	~380	
	Air-jet				~670	~390	
Flat PET	Ring	260 × 130 mm^2^; folded fabric	360 mL; 60 min; 40 °C; 5 cycles	Non-bio liquid detergent	0.04%	-	[29]
Textured PET	Ring				0.01%	-	
PET staple fiber	Ring				0.02%	-	
PET fiber	Vortex	290 × 150 mm^2^; hemmed fabric; 30 tex	360 mL; 45 min; 40 °C; 5 cycles	Without detergent	348.7	-	[30]
	Ring				459.79	-	
	Ring	290 × 150 mm^2^; hemmed fabric; 15 tex			276.03	-	
	Vortex				218.24	-	

MP: microplastic, PET: polyethylene terephthalate, LAS: linear alkylbenzene sulfonate, AATCC: The American Association of Textile Chemists and Colorists.

**Table 3:** Impact of dyeing and finishing on MP shedding during dyeing and washing.

Production Process	Sliver/Yarn/Fabric	Dye/Finish	Number of MPs Released (MPs/g)	References
During dyeing	Polyester sliver, 1.5 g	BEMACRON E-RD	Step 1: 86 ± 32 Step 2: 127 ± 27	[14]
	Polyester yarn, rotor, 1.5 g		797 ± 45 884 ± 154	
	Polyester yarn, air-jet, 1.5 g		80 ± 20 49 ± 2	
	Polyester yarn, ring, 1.5 g		43 ± 17 26 ± 12	
Washing (AATCC standard)	Knitted cotton fabric, ring, 10 × 10 cm	C.I. reactive blue 19	~9700	[8]
		Durable press	~14,200	
		Silicon softener	~11,900	
		Water repellent	~9300	

## Data Availability

No new data were created in this study.
